# Mobilization Started Within 2 Hours After Abdominal Surgery Improves Peripheral and Arterial Oxygenation: A Single-Center Randomized Controlled Trial

**DOI:** 10.1093/ptj/pzab094

**Published:** 2021-03-20

**Authors:** Anna Svensson-Raskh, Anna Regina Schandl, Agneta Ståhle, Malin Nygren-Bonnier, Monika Fagevik Olsén

**Affiliations:** 1 Department of Neurobiology, Care Sciences and Society, Division of Physiotherapy, Karolinska Institutet, Stockholm, Sweden; 2 Women’s Health and Allied Health Professionals Theme, Medical Unit Occupational Therapy and Physiotherapy, Karolinska University Hospital, Stockholm, Sweden; 3 Department of Molecular Medicine and Surgery, Karolinska Institutet, Stockholm, Sweden; 4 Department of Anesthesia and Intensive Care, Södersjukhuset, Stockholm, Sweden; 5 Department of Neuroscience and Physiology, Division of Health & Rehabilitation/Physical Therapy, Sahlgrenska Academy, Gothenburg University, Gothenburg, Sweden; 6 Department of Physiotherapy, Sahlgrenska University Hospital, Gothenburg, Sweden

**Keywords:** Early Ambulation, Physiotherapy, Postoperative Care, Postoperative Complications, Respiratory Function

## Abstract

**Objective:**

The aim of this study was to investigate if mobilization out of bed, within 2 hours after abdominal surgery, improved participants’ respiratory function and whether breathing exercises had an additional positive effect.

**Methods:**

Participants were 214 consecutively recruited patients who underwent elective open or robot-assisted laparoscopic gynecological, urological, or endocrinological abdominal surgery with an anesthetic duration of >2 hours. They were recruited to a randomized controlled trial. Immediately after surgery, patients were randomly assigned to 1 of 3 groups: mobilization (to sit in a chair) and standardized breathing exercises (n = 73), mobilization (to sit in a chair) only (n = 76), or control (n = 65). The interventions started within 2 hours after arrival at the postoperative recovery unit and continued for a maximum of 6 hours. The primary outcomes were differences in peripheral oxygen saturation (SpO_2_, as a percentage) and arterial oxygen pressure (PaO_2_, measured in kilopascals) between the groups. Secondary outcomes were arterial carbon dioxide pressure, spirometry, respiratory insufficiency, pneumonia, and length of stay.

**Results:**

Based on intention-to-treat analysis (n = 214), patients who received mobilization and breathing exercises had significantly improved SpO_2_ (mean difference [MD] = 2.5%; 95% CI = 0.4 to 4.6) and PaO_2_ (MD = 1.40 kPa; 95% CI = 0.64 to 2.17) compared with the controls. For mobilization only, there was an increase in PaO_2_ (MD = 0.97 kPa; 95% CI = 0.20 to 1.74) compared with the controls. In the per-protocol analysis (n = 201), there were significant improvements in SpO_2_ and PaO_2_ for both groups receiving mobilization compared with the controls. Secondary outcome measures did not differ between groups.

**Conclusion:**

Mobilization out of bed, with or without breathing exercises, within 2 hours after elective abdominal surgery improved SpO_2_ and PaO_2_.

**Impact:**

The respiratory effect of mobilization (out of bed) immediately after surgery has not been thoroughly evaluated in the literature. This study shows that mobilization out of bed following elective abdominal surgery can improve SpO_2_ and PaO_2_.

**Lay Summary:**

Mobilization within 2 hours after elective abdominal surgery, with or without breathing exercises, can improve patients’ respiratory function.

## Introduction

Early mobilization is recommended after abdominal surgery and is commonly considered to be one of the factors that enhance patients’ recovery. In the fast-track concept, Enhanced Recovery After Surgery, it is recommended that patients are mobilized on the day of surgery.^1^ However, adherence to these guidelines is unclear.^2^^,^^3^ Previous literature indicates that patients’ time out of bed in the first days after surgery is often short.^4–6^ Clinical practice for early mobilization at postoperative recovery units differs. Some patients are mobilized out of bed, some sit on the bedside, and some stay in bed with the bed head elevated. The frequency, duration, and intensity of early mobilization also vary in different settings.^3^

Postoperative pulmonary complications, such as atelectasis or pneumonia, are common after surgery and may cause unnecessary discomfort, prolonged hospital stay, and increased health care costs.^7^^,^^8^ Usually, postoperative pulmonary complications are consequences of reduced functional residual capacity (FRC) following surgery, anesthesia, and immobilization.^8–13^ Mobilization or being in an upright position and breathing exercises are assumed to prevent a reduction in FRC^14–16^ and are frequently recommended after surgery.^8^^,^^17–19^ Previous studies have evaluated mobilization combined with other treatments,^1^^,^^20^ such as in early mobilization protocols,^3^ with different breathing exercises,^21^^,^^22^ mobilization starting at postoperative day 1 (POD1),^3^^,^^18^^,^^23^^,^^24^ or compared with standard of care.^6^^,^^25^ To date, the isolated effect of immediate postoperative mobilization on respiratory function has not been thoroughly investigated.^3^^,^^18^

To our knowledge, no study has compared the immediate respiratory effect of mobilization out of bed to sitting in a chair plus breathing exercises, starting on the same day as abdominal surgery, with an untreated control group.

Therefore, a randomized controlled trial was conducted, hypothesizing that mobilization, within 2 hours after elective open or robot-assisted laparoscopic gynecological, urological, or endocrinological abdominal surgery, would improve patients’ respiratory function and that breathing exercises would have an additional positive effect.

## Methods

A 3-arm randomized controlled trial was conducted at a postoperative recovery unit at a university hospital in Stockholm, Sweden. The study was approved by the Regional Ethical Review Board in Stockholm and registered at ClinicalTrials.gov (NCT02929446).

### Participants

**Figure 1:**
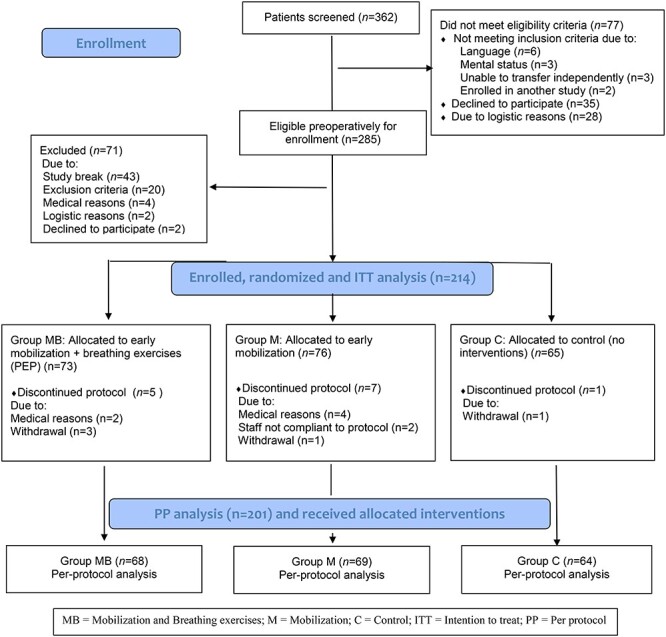
CONSORT flowchart of the study design. C = control; ITT = intention-to-treat; M = mobilization only; MB = mobilization and breathing exercises; PEP = positive expiratory pressure; PP = per-protocol.

Between January 23 and September 22, 2017, all adult Swedish- and English-speaking patients planned for elective gynecological, urological, or endocrinological open or robot-assisted laparoscopic surgery because of cancer, with an expected anesthetic duration ≥2 hours, were considered eligible for inclusion. Patients were excluded if they required assistance for mobilization before surgery, were not able to understand instructions, or enrolled in concurrent studies at the postoperative recovery unit. Patients were excluded prior to randomization if the surgical procedure prevented mobilization, the anesthesiologist in charge at the recovery unit considered the patient to be unfit for mobilization because of cardiorespiratory instability requiring immediate treatment, or if the patient arrived at the postoperative recovery unit after 6 pm (when no physical therapist was present) (Fig. 1).

### Procedure

Patients listed for abdominal surgery were required to attend a presurgical evaluation at an outpatient clinic approximately 2 weeks prior to surgery. Here, the research nurse identified eligible patients, and potential participants received verbal and written information about the study. Consenting patients were entered in the trial, and assessments of peripheral oxygen saturation (SpO_2_), forced vital capacity (FVC), forced expiratory volume in 1 second (FEV_1_), peak expiratory flow (PEF), and FEV_1_/FVC were performed. SpO_2_ was assessed with an oximeter (TuffSat Pulse Oximeter, GE Datex-Ohmeda, Inc, Frankfort, KY, USA) OK Spirometry was assessed with a portable microspirometer (Carefusion MicroLoop, Vyaire Medical Inc, Chatham Maritime, Kent, UK) performed according to a predefined standardized protocol.^26^

### Randomization and Masking

Immediately after surgery, when the patients entered the postoperative recovery unit, a nurse at the recovery unit, independent of the trial, randomly assigned the spontaneously breathing patients to mobilization and breathing exercises, mobilization only, or control.

A computer-generated randomization in blocks of 9 was used to allocate patients to the different trial groups (1:1:1). Treatment allocation was concealed by random selection of opaque sealed envelopes prepared by an investigator with no further involvement in the trial. It was not possible to mask patients or health care professionals at the recovery unit to treatment allocation. The surgeons who performed the operations, the anesthesiologists at the postoperative recovery unit, the research nurses, and the physical therapists who performed the postoperative spirometry were all masked with regard to group allocation. All data were coded, and group assignment was masked for analyses.

### Interventions

The intervention period started within 2 hours after arrival at the postoperative recovery unit. Interventions continued for 6 hours, or less if the anesthesiologist in charge considered the patient fit for discharge to the surgical ward. The 3 groups were mobilization and breathing exercises, mobilization only, and control.

#### Mobilization and Breathing Exercises

For this group, mobilization out of bed, assisted by a physical therapist and a nurse, to sit in a chair (or unsupported on the bedside if unable to stand and transfer to a chair) was performed. The patient was instructed to sit for as long as he or she could. If required, sitting was interspersed by 1 hour of bed rest with the bed head at ≥45 degrees. The patient was instructed by a physical therapist to perform 10 × 3 deep breathing exercises every hour with a positive expiratory pressure device (PEP T-piece and Resistor, Intersurgical AB, Danderyd, Sweden) at a mid-expiratory pressure of 10 to 15 cm of H_2_O. The physical therapist ensured that the breathing exercises were performed correctly.

#### Mobilization

For this group, mobilization out of bed, assisted by a physical therapist and a nurse, to sit in a chair (or unsupported on the bedside if unable to stand and transfer to a chair) was performed. The patient was instructed to sit as long as he or she could. If required, sitting was interspersed by 1 hour of bed rest with the bed head at ≥45 degrees.

#### Control

For the control group, no mobilization or breathing exercises were performed.

### Data Collection

Demographic variables such as weight, height, age, history of obstructive/restrictive respiratory disease, smoking, American Society of Anesthesiologists score,^27^ and planned surgery were collected from the patient and his or her medical records. The following treatment-related data were collected from the bedside chart at the recovery unit: time for arrival/discharge, type of surgery, perioperative anesthesia, and total length of anesthesia and surgery. At each hour, patients rated pain and nausea on a numeric rating scale, where 0 = no pain/nausea and 10 = the worst pain/nausea imaginable.^28^ The time points for the start of mobilization and the duration and frequency of mobilization were registered in a bedside chart.

### Outcome Measures

The primary outcomes were change (between groups and over time) in SpO_2_ (as a percentage) measured peripherally with a pulse oximeter and arterial oxygen pressure (PaO_2_, measured in kilopascals) measured via arterial blood gas sample. PaO_2_ could not be assessed for 15 patients because they did not receive an arterial line during surgery. Assessments were performed at arrival to the postoperative recovery unit and every subsequent hour until discharge. The oxygen supply was disconnected 15 minutes before each hourly assessment. The assessments at arrival were performed with all patients in a supine position. From every hour thereafter, only the outcome of the controls was measured in a supine position. For patients in the groups receiving mobilization, outcomes were measured with the patient sitting in the chair or, if in bed, with a bed head elevation of ≥45 degrees.

The secondary outcomes were changes (between groups and over time) in arterial carbon dioxide pressure (PaCO_2_, in kilopascals) assessed every hour from arrival to discharge from the recovery unit via arterial blood gas sample (any oxygen supply was disconnected 15 minutes prior to test). Changes in FVC, FEV_1_, PEF, and FEV_1_/FVC in the groups between preoperative baseline and POD1 were assessed with spirometry.^26^ Respiratory insufficiency, defined as an SpO_2_ of <90%, a PaO_2_ of <8 kPa, and/or PaCO_2_ of ≥6.5 kPa,^29^ was registered if present (at the hourly blood sample test) every hour from arrival to discharge from the postoperative recovery unit. Information about pneumonia and length of stay was retrieved from the medical record. Pneumonia was considered present if the patient had newly evolving chest radiograph infiltrate and 2 or more of the following criteria: temperature of >38.3°C, leukocyte count of >12,000 μL^−1^, and purulent sputum.^29^Supplementary File 1 displays the timeline of the study protocol.

### Adverse Effects

Possible adverse consequences of mobilization and breathing exercises, such as falls, drainage that accidentally fell out (extubated) during or due to the mobilization, and surgical wound ruptures, were monitored according to clinical procedures at the hospital.

#### Data Analysis

Assuming that SpO_2_ would increase by 2% (SD = 4) or that PaO_2_ would increase by 0.5 kPa (SD = 1),^30^ the number of patients required to establish 80% power at a significance level of 5% was 63 in each arm. Differences in patient characteristics between the 3 treatment groups were presented as means and SDs or as numbers and proportions where appropriate. Intention-to-treat and per-protocol analyses were conducted. The Student *t* test was used to compare the total time of mobilization between the group receiving mobilization and breathing exercises and the group receiving mobilization only. Repeated measures of SpO_2_, PaO_2_, and PaCO_2_ over 4 hours of mobilization were analyzed using a linear mixed model^31^ with the following factors: group (mobilization and breathing exercises, mobilization only, and control), time (1, 2, 3, and 4 hours), type of surgery (open or robot assisted), and the 2-way and 3-way interactions between the factors. Age and SpO_2_, PaO_2_, and PaCO_2_ at baseline were included as covariates.

Potential differences in FVC, FEV_1_, PEF, and FEV_1_/FVC between preoperative and postoperative measurements at POD1 were analyzed using a linear mixed model^31^ including the following factors: group time (baseline, POD1), type of surgery, and the 2-way and 3-way interactions between the factors; age and body mass index were covariates. Results were presented as mean differences (MDs) with 95% CIs. Bonferroni adjustments were used to correct for multiple comparisons.

For respiratory insufficiency^29^ and pneumonia,^29^ logistic regression was used to assess the association between respiratory insufficiency and the interventions (mobilization and breathing exercises, mobilization only, and control) and the association between pneumonia and the interventions (mobilization and breathing exercises, mobilization only, and control), respectively. Results were presented as odds ratios (ORs) with 95% CIs. The models were adjusted for age (<61 years, 61–74 years, and >74 years),^10^^,^^32^ sex, body mass index (<30 or ≥30),^10^ smoking (never/ever),^10^ preoperative lung disease (restrictive and/or obstructive diseases, chronic obstructive pulmonary disease, obstructive sleep apnea, or asthma registered in the medical records) (yes or no),^10^^,^^32^ duration of anesthesia (<4 hours, ≥4 hours),^7^^,^^32^ and type of surgery (open or robotic surgery).^10^^,^^32^

For length of stay at the recovery unit and at the hospital, the 3 treatment groups were compared using analysis of variance. A *P* value of <.05 was considered statistically significant. The Statistical Package for the Social Sciences Version 24 (SPSS; IBM Corp, Armonk, NY, USA) was used for all statistical analyses.

## Role of the Funding Source

The funding source had no role in the design, conduct, or reporting of this trial.

## Results

Between January 23 and September 22, 2017, a total of 362 patients were screened for inclusion. Among the 285 eligible patients, a total of 71 were excluded. Patients were excluded because of an unplanned study break during the summer, when no research nurse was present (n = 43); contraindication of postoperative mobilization (n = 20); severe cardiorespiratory problems during surgery (medical reasons) (n = 4); arrival at the postoperative recovery unit after 6 pm, when no physical therapist was on duty (logistic reasons) (n = 2); and declined participation (n = 2) (Fig. 1).

The remaining 214 patients were randomly assigned to 1 of the 2 groups receiving mobilization or the control group at arrival to the postoperative unit. Seventy-three patients were assigned to mobilization and breathing exercises, 76 were assigned to mobilization only, and 65 were assigned to control. Six patients had cardiorespiratory instability prior to trial start and could not fulfill mobilization. For 2 patients, the caregivers did not adhere to the study protocol, and 5 patients withdrew from the study (Fig. 1).

Table 1 shows the characteristics and demographics of the 214 enrolled patients. Approximately 60% were women. The most common types of surgery were robot-assisted laparoscopic urologic surgery (36%), such as prostatectomy or cystectomy (radical with construction of orthotopic bladder substitution), and open gynecological surgery (27%), mostly for ovarian cancer stages III and IV, involving bowel surgery (colorectal surgery), and stripping of the diaphragm.

**Table 1:** Demographic and Perioperative Characteristics of the Intention-to-Treat Population by Study Group (n = 214)^a^

**Variable**	**Mobilization and Breathing Exercises (n = 73)**	**Mobilization Only** **(n = 76)**	**Control (n = 65)**
Sex			
Women	45 (62)	44 (58)	41 (63)
Men	28 (38)	32 (42)	24 (37)
Age, y, median (IQR)	72 (63.5–77)	69 (60–73)	68 (59–72)
BMI, kg/m^2^, mean (SD)	27 (6.3)	28.2 (5.9)	26.3 (4.4)
ASA physical status			
1	1 (1)	9 (12)	12 (18)
2	48 (66)	46 (59)	36 (55)
≥3	25 (33)	22 (29)	17 (28)
Smoking status*^b^*			
Never smoked	37 (51)	42 (55)	33 (51)
Former smoker	29 (40)	29 (38)	24 (37)
Current smoker	7 (9)	5 (7)	8 (12)
Respiratory disease*^c^*	15 (21)	20 (26)	23 (35)
Preoperative SpO_2_, %	97.2 (1.5)	97.2 (1.6)	97.7 (1.4)
Type of surgery			
Gynecological open	20 (27)	20 (26)	18 (28)
Gynecological robot-assisted laparoscopic	17 (23)	14 (18)	12 (18)
Urological open	0 (0)	1 (1)	2 (3)
Urological robot-assisted laparoscopic	26 (36)	30 (40)	24 (37)
Sarcoma and NET	9 (12)	8 (11)	9 (14)
Adrenalectomy	1 (1)	3 (4)	0 (0)
Type of perioperative anesthesia			
General	13 (18)	13 (17)	9 (14)
General and spinal	35 (48)	40 (53)	32 (49)
General and epidural	25 (34)	23 (30)	24 (37)
Perioperative bleeding, mL, mean (SD)	292 (607)	343 (788)	210 (311)
Length of surgery, h:min, mean (SD)	2:5 (1.4)	3:0 (1.3)	2:5 (1.2)
Length of anesthesia, h:min, mean (SD)	4:1 (1.4)	4:3 (1.4)	4:1 (1.3)

*^a^* Data are numbers (percentages) of patients unless otherwise indicated. ASA = American Society of Anesthesiologists; BMI = body mass index; IQR = interquartile range; NET = neuroendocrine tumors; SpO_2_ = peripheral oxygenation (oxygen saturation).

*^b^* Former smoker = ceased smoking >6 months before preoperative assessment; current smoker = smoking tobacco regularly within 6 months of assessment.

*^c^* Respiratory disease = (in the medical journal) a physician’s note about restrictive and/or obstructive diseases, such as chronic obstructive pulmonary disease, obstructive sleep apnea, or asthma.

### Intervention Compliance

Of the 214 randomized patients, 201 patients completed the allocated intervention. The mean mobilization time was 105 minutes, ranging from 10 to 265 minutes, with no statistically significant difference between the 2 groups receiving mobilization (*P* = .93). All patients in the group receiving mobilization and breathing exercises completed the breathing exercises according to protocol.

Four patients, 1 in the group receiving mobilization and breathing exercises and 3 in the group receiving mobilization only, sat unsupported on the bedside. They were unable to mobilize to a chair because they lacked sufficient leg muscle tonus as a result of spinal/epidural anesthesia or low blood pressure and dizziness, which made it unsafe to stand up.

### Primary Outcomes

**Figure 2:**
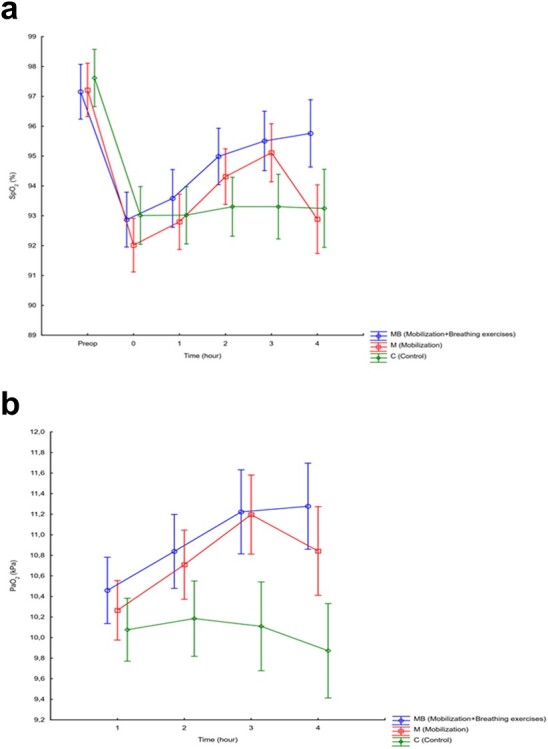

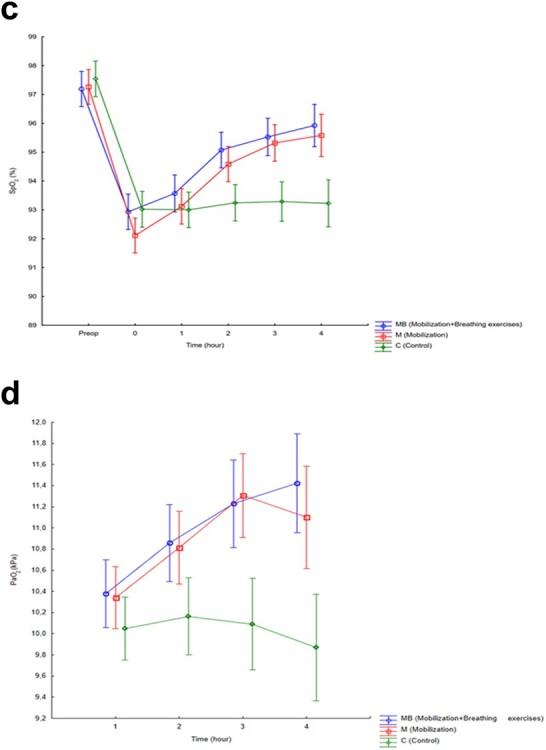
(a and b) Changes in primary outcomes (peripheral oxygen saturation [SpO_2_], as a percentage, and arterial oxygen pressure [PaO_2_], in kilopascals) across time points, by treatment groups, at 95% CIs, in the intention-to-treat population (n = 214). Changes in SpO_2_ (as a percentage) and PaO_2_ (in kilopascals) across time points, by treatment groups, at 95% CIs, in the per-protocol population (n = 201). Preop = preoperatively (c and d).

There were no baseline differences in SpO_2_ (Tab. 1). In the intention-to-treat analysis (n = 214), patients who received mobilization only did not improve in SpO_2_ (MD = −0.36%; 95% CI = −2.49 to 1.77; *P* > .99) compared with the controls. SpO_2_ improved significantly with time in patients who received mobilization and breathing exercises (MD = 2.5%; 95% CI = 0.4 to 4.6; *P* = .01) compared with the controls. There was a significant improvement in PaO_2_ for patients who received mobilization only (MD = 0.97 kPa; 95% CI = 0.20 to 1.74; *P* = .009) and patients who received mobilization and breathing exercises (MD = 1.40 kPa; 95% CI = 0.64 to 2.17; *P* < .001) compared with the controls. Changes in PaO_2_ did not differ between patients who were assigned mobilization and breathing exercises and patients who were assigned mobilization only (MD = 0.44 kPa; 95% CI = −0.31 to 1.17; *P* = .47) (Fig. 2; Tab. 2; Suppl. File 2).

**Table 2:** Primary and Secondary Outcomes Presented as Mean Score Differences Between Groups in the ITT (n = 214) and PP (n = 201) Populations^a^

**Outcomes**	**Time (h)**	**Mean Score Differences (95% CIs) Between Groups**					
		**ITT Population**			**PP Population**		
		**Mobilization and Breathing Exercises: Control**	**Mobilization Only: Control**	**Mobilization and Breathing Exercises: Mobilization Only**	**Mobilization and Breathing Exercises: Control**	**Mobilization Only: Control**	**Mobilization and Breathing Exercises: Mobilization Only**
Primary							
SpO_2_	Preoperatively	−0.46 (−2.09 to 1.17)	−0.40 (−2.00 to 1.20)	−0.06 (−1.63 to 1.51)	−0.35 (−1.42 to 0.72)	−0.28 (−1.33 to 0.77)	−0.07 (−1.12 to 0.99)
	0	−0.14 (−1.77 to 1.49)	−0.99 (−2.60 to 0.61)	0.86 (−0.71 to 2.43)	−0.09 (−1.16 to 0.98)	−0.91 (−1.96 to 0.14)	0.82 (−0.23 to 1.88)
	1	0.56 (−1.11 to 2.23)	−0.22 (−1.85 to 1.40)	0.78 (−0.86 to 2.43)	0.57 (−0.52 to 1.66)	0.12 (−0.94 to 1.18)	0.45 (−0.64 to 1.54)
	2	1.68 (0.01 to 3.36)*^b^*	1.01 (−0.65 to 2.66)	0.68 (−0.95 to 2.30)	1.83 (0.74 to 2.91)*^b^*	1.34 (0.28 to 2.41)*^b^*	0.49 (−0.58 to 1.55)
	3	2.20 (0.40 to 4.00)*^b^*	1.81 (0.03 to 3.58)*^b^*	0.40 (−1.31 to 2.10)	2.24 (1.08 to 3.39)*^b^*	2.03 (0.89 to 3.17)*^b^*	0.21 (−0.96 to 1.32)
	4	2.51 (0.40 to 4.62)*^b^*	−0.36 (−2.49 to 1.77)	2.87 (0.90 to 4.84)	2.70 (1.36 to 4.04)*^b^*	2.36 (1.02 to 3.69)*^b^*	0.35 (−0.92 to 1.61)
PaO_2_	1	0.38 (−0.16 to 0.93)	0.19 (−0.33 to 0.70)	0.19 (−0.34 to 0.73)	0.33 (−0.21 to 0.87)	0.29 (−0.22 to 0.80)	0.04 (−0.50 to 0.57)
	2	0.65 (0.02 to 1.29)*^b^*	0.53 (0.08 to 1.13)	0.13 (−0.48 to 0.74)	0.69 (0.06 to 1.33)*^b^*	0.65 (0.04 to 1.26)*^b^*	0.04 (−0.57 to 0.66)
	3	1.13 (0.38 to 1.84)*^b^*	1.09 (0.38 to 1.80)*^b^*	0.03 (−0.66 to 0.72)	1.14 (0.40 to 1.87)*^b^*	1.21 (0.50 to 1.93)*^b^*	−0.08 (−0.78 to 0.63)
	4	1.41 (0.64 to 2.17)*^b^*	0.97 (0.20 to 1.74)*^b^*	0.44 (−0.31 to 1.17)	1.55 (0.71 to 2.40)*^b^*	1.23 (0.37 to 2.09)*^b^*	0.32 (−0.50 to 1.15)
Secondary							
PaCO_2_	1	−0.12 (−0.33 to 0.09)	−0.02 (−0.22 to 0.18)	−0.10 (−0.30 to 0.11)	−0.13 (−0.34 to 0.09)	−0.02 (−0.22 to 0.18)	−0.11 (−0.32 to 0.11)
	2	−0.22 (−0.43 to −0.01)*^b^*	−0.19 (−0.39 to 0.01)	−0.03 (−0.23 to 0.18)	−0.23 (−0.44 to −0.02)*^b^*	−0.22 (−0.42 to −0.02)*^b^*	−0.01 (−0.22 to 0.19)
	3	−0.13 (−0.35 to 0.09)	−0.05 (−0.26 to 0.16)	−0.08 (−0.29 to 0.13)	−0.15 (−0.37 to 0.07)	−0.07 (−0.29 to 0.14)	−0.08 (−0.29 to 0.14)
	4	−0.12 (−0.37 to 0.12)	0.07 (−0.17 to 0.32)	−0.20 (−0.43 to 0.04)	−0.15 (−0.39 to 0.10)	0.05 (−0.19 to 0.30)	−0.20 (−0.44 to 0.04)

*^a^* Covariates were as follows: for peripheral oxygen saturation (SpO_2_): age; for arterial oxygen pressure (PaO_2_): age and PaO_2_ at time 0; for arterial carbon dioxide pressure (PaCO_2_): age and PaCO_2_ at time 0. ITT = intention-to-treat; PP = per-protocol.

*^b^* Statistically significant at *P* < .05. Adjustment for multiple comparisons: Bonferroni.

There were similar results for PaO_2_ in the per-protocol analysis (n = 201). For SpO_2_, there were significant improvements for mobilization only (MD = 2.4%; 95% CI = 1.02 to 3.70; *P* < .001), and for mobilization and breathing exercises (MD = 2.7%; 95% CI = 1.36 to 4.04; *P* < .001) compared with controls (Tab. 2; Fig. 2). PaO_2_ reached its peak value within 3 hours after the start of the trial in the 2 groups receiving mobilization. Four hours after surgery, PaO_2_ had improved significantly for patients who completed mobilization and breathing exercises (MD = 1.6 kPa; 95% CI = 0.71 to 2.40; *P* < .001) and for patients who completed mobilization only (MD = 1.2 kPa; 95% CI = 0.37 to 2.09; *P* = .002) compared with the controls. Detailed information on raw mean score results for the intention-to-treat and per-protocol analyses can be found in Supplementary File 2. The type of surgery (open or robot assisted) did not influence changes in SpO_2_ (*P* = .26) or PaO_2_ (*P* = .58).

### Secondary Outcomes

PaCO_2_ decreased over time in the 3 groups (*P* < .001), with no differences (*P* = .07 for intention-to-treat analysis and *P* = .06 for per-protocol analysis) between the groups (Tab. 2; Suppl. File 2). There were reductions in FVC, FEV_1_, and PEF between preoperative baseline and POD1 (*P* < .001) in the 3 groups (Tab. 3; Suppl. File 3).

**Table 3:** Secondary Outcomes in the Intention-to-Treat Population (n = 214) by Treatment Group^a^

**Secondary Outcomes**	**Mobilization and Breathing Exercises (n = 73)**	**Mobilization Only (n = 76)**	**Control** **(n = 65)**	***P*** ^***b***^
FVC				
Preoperatively	3.36 (3.16 to 3.57)	3.21 (3.01 to 3.40)	3.09 (2.88 to 3.31)	
POD1	2.85 (2.62 to 3.07)	2.67 (2.45 to 2.89)	2.50 (2.27 to 2.71)	.81
MD (95% CI)	−0.51*^c^* (−0.67 to −0.35)	−0.54*^c^* (−0.70 to −0.38)	−0.59*^c^* (−0.76 to −0.42)	
FEV_1_				
Preoperatively	2.53 (2.37 to 2.70)	2.42 (2.26 to 2.58)	2.39 (2.22 to 2.56)	
POD1	2.17 (1.99 to 2.35)	2.06 (1.89 to 2.24)	1.96 (1.77 to 2.14)	.67
MD (95% CI)	−0.36*^c^* (−0.49 to −0.23)	−0.35*^c^* (−0.48 to −0.22)	−0.43*^c^* (−0.57 to −0.29)	
PEF				
Preoperatively	376.95 (347.59 to 406.32)	375.56 (347.27 to 403.84)	366.68 (336.01 to 397.36)	
POD1	312.52 (279.52 to 345.52)	308.05 (276.12 to 340.91)	302.80 (268.11 to 337.48)	.89
MD (95% CI)	−64.44*^c^* (−90.79 to −38.09)	−67.04*^c^* (−93.15 to −40.94)	−63.88*^c^* (−91.86 to −35.90)	
FEV_1_/FVC				
Preoperatively	77.61 (75.65 to 79.66)	78.14 (76.16 to 80.11)	78.35 (76.21 to 80.49)	
POD1	78.89 (76.54 to 81.24)	77.63 (75.32 to 79.94)	79.67 (77.19 to −82.14)	.36
MD (95% CI)	1.28 (−0.75 to 3.32)	−0.51 (−2.52 to 1.50)	1.31 (−0.85 to 3.47)	

*^a^* Data are reported as mean scores (95% CIs) unless otherwise indicated. FEV_1_ = forced expiratory volume in 1 second; FVC = forced vital capacity in L; MD = mean difference; PEF = peak expiratory flow in L/min; POD1 = postoperative day 1.

*^b^* Difference between the groups; a *P* value of less than .05 was considered statistically significant.

*^c^* Statistically significant difference (*P* < .05) within the group between preoperative assessment and POD1 assessment.

Respiratory insufficiency^29^ was found in 15 patients (21%) in the group receiving mobilization and breathing exercises, 25 (33%) in the group receiving mobilization only, and 17 (26%) in the control group, while pneumonia^29^ was present in 1, 3, and 5 patients in these groups, respectively. Compared with the controls, there was not sufficient evidence to determine a reduction in risk for respiratory insufficiency in patients who received mobilization and breathing exercises (OR = 0.52; 95% CI = 0.22 to 1.22) or mobilization only (OR = 1.14; 95% CI = 0.53 to 2.46) or for pneumonia in patients who received mobilization and breathing exercises (OR = 0.11; 95% CI = 0.01 to 1.14) or mobilization only (OR = 0.44; 95% CI = 0.09 to 2.08) (Tab. 4).

**Table 4:** Unadjusted and Adjusted Logistic Regression Showing Associations Between Interventions and Conditions in the Intention-to-Treat Population (n = 214)

**Condition**	**Group**	**Crude Model**		**Adjusted Model**	
		**Odds Ratio (95% CI)**	***P***	**Odds Ratio (95% CI)**	***P***
Respiratory insufficiency	Control (reference)	1.0			
	Mobilization and breathing exercises	0.73 (0.33 to 1.61)	.44	0.52 (0.22 to 1.22)	.13
	Mobilization only	1.38 (0.66 to 2.88)	.38	1.14 (0.53 to 2.46)	.74
Pneumonia	Control (reference)	1.0			
	Mobilization and breathing exercises	0.17 (0.02 to 1.47)	.11	0.11 (0.01 to 1.14)	.06
	Mobilization only	0.49 (0.11 to 2.15)	.35	0.44 (0.09 to 2.08)	.3

The mean lengths of stay at the postoperative recovery unit were 12 hours (SD = 10), 10 hours (SD = 9), and 9 hours (SD = 7) for mobilization and breathing exercises, mobilization only, and control, respectively (*P = .*63). The total lengths of hospital stay did not differ between the groups: 5 days (SD = 3) for mobilization and breathing exercises, 4 days (SD = 3) for mobilization and breathing exercises, and 4 days (SD = 3) for control (*P* = .42).

Similar ratings of pain and nausea were seen in all 3 groups, with no statistically significant differences between the groups. Pain decreased over time (*P* < .001), and nausea was constant over time (*P* = .60). The mean values of pain were 1.1 (SD = 1.6), 1.0 (SD = 1.3), and .9 (SD = 1.4) for mobilization and breathing exercises, mobilization only, and control, respectively. The mean values of nausea were 0.7 (SD = 1.2), 0.9 (SD = 1.4), and 0.5 (SD = 1.0) for mobilization and breathing exercises, mobilization only, and control, respectively.

The patients experienced no adverse effects while being mobilized.

## Discussion

To our knowledge, this is the first study evaluating the respiratory effects of immediate postoperative mobilization from bed to sitting in a chair within 2 hours after surgery, compared with the controls. The results confirmed the hypothesis that mobilization immediately after elective, open or robot-assisted laparoscopic gynecological, urological, and endocrinological surgery can improve SpO_2_ and PaO_2_.

Immediately after surgery, SpO_2_ decreased in all groups compared with preoperative baseline.

It is well known that oxygenation decreases after surgery.^33^ However, during the intervention period, SpO_2_, and PaO_2_ improved in both groups receiving mobilization compared with the control group (Fig. 2, A and C). It is reasonable to assume that if oxygenation returns to normal values, perhaps due to a normalization of FRC and a lack of atelectasis, patients would have better respiratory status at discharge to surgical wards, without a need for additional oxygen.

Earlier trials in other populations and in healthy participants suggest that changing position, to sitting in a chair or to standing, is a simple way to achieve an increased lung volume, provide inspiratory flow in small airways, and prevent and treat atelectasis.^14^^,^^15^^,^^17^^,^^34^^,^^35^ The effect on volume is instantaneous, but when returning to bed, respiration returns to lower volume. Nevertheless, there are effects that persist. Recruited airways are less likely to re-collapse when opened, which can affect SpO_2_ and PaO_2._^33^^,^^36^ In the early postoperative phase, when patients are bedbound, they are often in a position with more or less elevated bed head. However, sitting up in a chair or unsupported on the bedside has an advantage to sitting in bed as it prevents patients slouching or slumping—a position that reduces the tidal volume and subsequently the FRC, to the same volumes as in supine.^14^

In present trial, the effects of additional breathing exercises were also evaluated. In the per-protocol analysis, the respiratory effects on SpO_2_ and PaO_2_ were similar for patients with mobilization and breathing exercises as for mobilization only. Mobilization as an intervention seems to be sufficient for patients undergoing elective open or robot-assisted laparoscopic gynecological, urological, or endocrinological abdominal surgery. It is possible that the time spent sitting had sufficient impact on the respiratory system and that the breathing exercises did not confer additional improvement in respiratory function. Some patients are at a higher risk of developing postoperative pulmonary complications because of comorbidities, advanced age, prolonged surgery, or surgery close to the diaphragm,^10^^,^^32^ and supervised breathing exercises may be beneficial.^9^^,^^37^ However, there is no consensus concerning which breathing exercise are superior in postoperative care.^38^^,^^39^ Even though mobilization has been shown to increase FRC,^14^^,^^15^^,^^36^ an additional increase created by positive expiratory pressure may decrease the atelectatic areas in the lungs.^21^^,^^40^ In other studies where breathing exercises were combined with mobilization, postoperative oxygenation, tidal volumes, and FRC increased,^21^^,^^25^ which theoretically could be sufficient to prevent pneumonia. In the present study, fewer patients in the groups receiving mobilization developed pneumonia compared with the controls, but the difference was not significant. However, Fernandez-Bustamante et al^7^ concluded that even mild postoperative pulmonary complications (atelectasis and long-term oxygen therapy) deserve increased attention and interventions so that perioperative outcomes can be improved, as they are associated with increased early postoperative mortality, intensive care unit intake, and total hospital stay.

There was a reduction in FVC, FEV_1_, and PEF from baseline to POD1 in all groups, which supports previous research.^25^^,^^41^ However, spirometry measurements were performed the day after the interventions, which might explain why there were no differences between the groups.

Between 20% and 30% of the patients in our study had respiratory sufficiency,^29^ which may be considered high compared with other studies.^42^ However, all assessments were performed after patients had been without oxygen supply for 15 minutes.

Previous studies have shown that atelectasis occurs already during induction of anesthesia and was greatest 2 hours after surgery. In addition, the atelectasis remained almost the same for 2 days postoperatively.^11^^,^^43^ In present study, we started the interventions within 2 hours of arriving at the recovery unit. Despite the relatively short intervention, SpO_2_ and PaO_2_ increased over time in the 2 groups receiving mobilization but decreased accordingly in the control group. Although the differences between the groups in SpO_2_ and PaO_2_ can be considered marginal, it may have a greater clinical significance. Our clinical experience is that patients who fall in SpO_2_/PaO_2_ postoperatively initially often receive oxygen as a first treatment, not mobilization. Our opinion is that this risks masking the patient’s true respiratory status. In the present study, all assessments of SpO_2_ and PaO_2_ were without oxygen, and this is the first study, to our knowledge, examining the isolated effect of mobilization (already within 2 hours after abdominal surgery) on respiratory function, such as SpO_2_ and PaO_2_. It might be considered too early to mobilize a patient out of bed within 2 hours after abdominal surgery. However, in the present study, we found that it was feasible, and we did not encounter any adverse consequences attributable to the intervention. There were no cases of falls with injury, drainage that fell out, or rupture of surgical wounds. In addition, mobilization out of bed immediately after abdominal surgery has been shown to affect patients’ physical and mental well-being. Patients report that they wake up, feel more alert, regain their autonomy, recover appetite and thirst, and feel that it is easier to breathe when they sit up in a chair compared with when they lie in bed.^44^

### Strengths and Limitations

The major strengths of this trial are the design, which limits the risk of selection bias, the inclusion of a relatively large sample of patients from a clinical setting, and the untreated control group, which allows investigation of the isolated effect of mobilization. There are also some limitations that need to be considered.

There were some baseline imbalances in patient characteristics. This was adjusted for in the mixed model but should be kept in mind when interpreting the results.

By limiting study inclusion to daytime only, there might be a risk that patients most susceptible for respiratory insufficiency have been excluded. Another limitation is that there was no group that performed only the breathing exercises. Therefore, the isolated effect of breathing exercises cannot be evaluated. Masking of the intervention for physical therapists, nurses, and patients at the postoperative recovery unit was not possible; however, the surgeons and anesthesiologists responsible for patient discharge from the postoperative recovery unit and the investigators who performed the outcome spirometry were masked with regard to group allocation. The patients in the control group were assessed in a supine position, but between the assessments the bed head could sometimes be positioned in 0 to 30 degrees, which is according to the clinical care at our unit.

For clinical reasons, not all patients had an arterial line during surgery. Therefore, there were missing PaO_2_ data. However, the analysis of SpO_2_ and PaO_2_ correlated well. Another important factor is that some patients were discharged before the third hour assessment, which might have reduced the power for these analyses. However, there were statistically significant differences between the groups. After discharge from postoperative care, the patients were treated according to standard care; therefore, the variables collected during this period were not controlled for and other confounding factors might have influenced the results. Length of stay at the recovery unit is not a precise outcome measure, and many factors influence the discharge decision. Patients were discharged from the postoperative recovery unit based on predefined criteria and not only according to respiratory status. Other important factors, such as pain, nausea, or prearranged overnight stay, also prolonged the stay. Thus, further study is needed to determine the ideal type, level, duration, and frequency of mobilization after surgery and the long-term respiratory effect.

Mobilization out of bed to sit in a chair, with or without breathing exercises, within 2 hours after surgery improved PaO_2_ and SpO_2_ after open or robot-assisted laparoscopic gynecological, urological, and endocrinological surgery without major side effects.

## Supplementary Material

SUPPLEMENTARY_1_pzab094

SUPPLEMENTARY_2_pzab094

SUPPLEMENTARY_3_pzab094

## Clinical Trial Registration

This study is registered at ClinicalTrials.gov (NCT02929446).

## References

[1] Ljungqvist O, Scott M, Fearon KC. Enhanced recovery after surgery: a review. JAMA Surg. 2017;152:292–298. 28097305 10.1001/jamasurg.2016.4952

[2] Gustafsson UO, Hausel J, Thorell A, Ljungqvist O, Soop M, Nygren J. Adherence to the enhanced recovery after surgery protocol and outcomes after colorectal cancer surgery. Arch Surg. 2011;146:571–577. 21242424 10.1001/archsurg.2010.309

[3] Castelino T, Fiore JF Jr, Niculiseanu P, Landry T, Augustin B, Feldman LS. The effect of early mobilization protocols on postoperative outcomes following abdominal and thoracic surgery: a systematic review. Surgery. 2016;159:991–1003. 26804821 10.1016/j.surg.2015.11.029

[4] Porserud A, Aly M, Nygren-Bonnier M, Hagstromer M. Objectively measured mobilisation is enhanced by a new behaviour support tool in patients undergoing abdominal cancer surgery. Eur J Surg Oncol. 2019;45:1847–1853. 31030805 10.1016/j.ejso.2019.04.013

[5] Browning L, Denehy L, Scholes RL. The quantity of early upright mobilisation performed following upper abdominal surgery is low: an observational study. Aust J Physiother. 2007;53:47–52. 17326738 10.1016/s0004-9514(07)70061-2

[6] Fiore JF, Castelino T, Pecorelli N, et al. Randomized controlled trial of an intervention to facilitate early mobilization after colorectal surgery: impact on postoperative pulmonary function and complications. Eur Respir J. 2017;50;OA1768.

[7] Fernandez-Bustamante A, Frendl G, Sprung J, et al. Postoperative pulmonary complications, early mortality, and hospital stay following noncardiothoracic surgery: a multicenter study by the perioperative research network investigators. JAMA Surg. 2017;152:157–166. 27829093 10.1001/jamasurg.2016.4065 PMC5334462

[8] Miskovic A, Lumb AB. Postoperative pulmonary complications. Br J Anaesth. 2017;118:317–334. 28186222 10.1093/bja/aex002

[9] Gallart L, Canet J. Post-operative pulmonary complications: understanding definitions and risk assessment. Best Pract Res Clin Anaesthesiol. 2015;29:315–330. 26643097 10.1016/j.bpa.2015.10.004

[10] Smetana GW, Lawrence VA, Cornell JE. Preoperative pulmonary risk stratification for noncardiothoracic surgery: systematic review for the American College of Physicians. Ann Intern Med. 2006;144:581–595. 16618956 10.7326/0003-4819-144-8-200604180-00009

[11] Hedenstierna G . Oxygen and anesthesia: what lung do we deliver to the post-operative ward? Acta Anaesthesiol Scand. 2012;56:675–685. 22471648 10.1111/j.1399-6576.2012.02689.x

[12] Craig DB . Postoperative recovery of pulmonary function. Anesth Analg. 1981;60:46–52. 7006464

[13] Duggan M, Kavanagh BP. Pulmonary atelectasis: a pathogenic perioperative entity. Anesthesiology. 2005;102:838–854. 15791115 10.1097/00000542-200504000-00021

[14] Jenkins SC, Soutar SA, Moxham J. The effects of posture on lung volumes in normal subjects and in patients pre-and post-coronary artery surgery. Physiotherapy. 1988;74:492–496.

[15] Hsu HO, Hickey RF. Effect of posture on functional residual capacity postoperatively. Anesthesiology. 1976;44:520–521. 1275319 10.1097/00000542-197606000-00010

[16] Fagevik Olsen M, Lannefors L, Westerdahl E. Positive expiratory pressure - common clinical applications and physiological effects. Respir Med. 2015;109:297–307. 25573419 10.1016/j.rmed.2014.11.003

[17] Mynster T, Jensen LM, Jensen FG, Kehlet H, Rosenberg J. The effect of posture on late postoperative oxygenation. Anaesthesia. 1996;51:225–227. 8712320 10.1111/j.1365-2044.1996.tb13637.x

[18] Nielsen KG, Holte K, Kehlet H. Effects of posture on postoperative pulmonary function. Acta Anaesthesiol Scand. 2003;47:1270–1275. 14616326 10.1046/j.1399-6576.2003.00240.x

[19] Ramos Dos Santos PM, Aquaroni Ricci N, Aparecida Bordignon Suster E, de Moraes Paisani D, Dias Chiavegato L. Effects of early mobilisation in patients after cardiac surgery: a systematic review. Physiotherapy. 2017;103:1–12. 27931870 10.1016/j.physio.2016.08.003

[20] Gustafsson UO, Scott MJ, Schwenk W, et al. Guidelines for perioperative care in elective colonic surgery: enhanced recovery after surgery (ERAS((R))) society recommendations. World J Surg. 2013;37:259–284. 23052794 10.1007/s00268-012-1772-0

[21] Ricksten SE, Bengtsson A, Soderberg C, Thorden M, Kvist H. Effects of periodic positive airway pressure by mask on postoperative pulmonary function. Chest. 1986;89:774–781. 3519107 10.1378/chest.89.6.774

[22] Silva YR, Li SK, Rickard MJ. Does the addition of deep breathing exercises to physiotherapy-directed early mobilisation alter patient outcomes following high-risk open upper abdominal surgery? Cluster randomised controlled trial. Physiotherapy. 2013;99:187–193. 23206316 10.1016/j.physio.2012.09.006

[23] Mackay MR, Ellis E, Johnston C. Randomised clinical trial of physiotherapy after open abdominal surgery in high risk patients. Aust J Physiother. 2005;51:151–159. 16137240 10.1016/s0004-9514(05)70021-0

[24] Wolk S, Linke S, Bogner A, et al. Use of activity tracking in major visceral surgery-the enhanced perioperative mobilization trial: a randomized controlled trial. J Gastrointest Surg. 2019;23:1218–1226. 30298422 10.1007/s11605-018-3998-0

[25] Fagevik Olsen M, Hahn I, Nordgren S, Lonroth H, Lundholm K. Randomized controlled trial of prophylactic chest physiotherapy in major abdominal surgery. Br J Surg. 1997;84:1535–1538. 9393272 10.1111/j.1365-2168.1997.02828.x

[26] Miller MR, Hankinson J, Brusasco V, et al. Standardisation of spirometry. Eur Respir J. 2005;26:319–338. 16055882 10.1183/09031936.05.00034805

[27] Fitz-Henry J . The ASA classification and peri-operative risk. Ann R Coll Surg Engl. 2011;93:185–187. 21477427 10.1308/147870811X565070a PMC3348554

[28] Williamson A, Hoggart B. Pain: a review of three commonly used pain rating scales. J Clin Nurs. 2005;14:798–804. 16000093 10.1111/j.1365-2702.2005.01121.x

[29] Jammer I, Wickboldt N, Sander M, et al. Standards for definitions and use of outcome measures for clinical effectiveness research in perioperative medicine: European Perioperative Clinical Outcome (EPCO) definitions: a statement from the ESA-ESICM joint taskforce on perioperative outcome measures. Eur J Anaesthesiol. 2015;32:88–105. 25058504 10.1097/EJA.0000000000000118

[30] Urell C, Emtner M, Hedenstrom H, Tenling A, Breidenskog M, Westerdahl E. Deep breathing exercises with positive expiratory pressure at a higher rate improve oxygenation in the early period after cardiac surgery--a randomised controlled trial. Eur J Cardiothorac Surg. 2011;40:162–167. 21146420 10.1016/j.ejcts.2010.10.018

[31] Brown H . Applied Mixed Models In Medicine. 3rd ed. Chichester, UK: Wiley; 2015.

[32] Neto AS, da Costa LGV, Hemmes SNT, et al. The LAS VEGAS risk score for prediction of postoperative pulmonary complications: an observational study. Eur J Anaesthesiol. 2018;35:691–701. 29916860 10.1097/EJA.0000000000000845 PMC7450515

[33] Marshall BE, Wyche MQ Jr. Hypoxemia during and after anesthesia. Anesthesiology. 1972;37:178–209. 4559455 10.1097/00000542-197208000-00009

[34] Moreno F, Lyons HA. Effect of body posture on lung volumes. J Appl Physiol. 1961;16:27–29. 13772524 10.1152/jappl.1961.16.1.27

[35] Blair E, Hickam JB. The effect of change in body position on lung volume and intrapulmonary gas mixing in normal subjects. J Clin Invest. 1955;34:383–389. 14354008 10.1172/JCI103086 PMC438639

[36] Lumb AB . Nunn’s Applied Respiratory Physiology. 8th ed. Edinburgh, UK: Elsevier; 2016.

[37] Olsén MF . Chest physical therapy in surgery: a theoretical model about who to treat. Breathe. 2005;1:308–314.

[38] Lawrence VA, Cornell JE, Smetana GW. Strategies to reduce postoperative pulmonary complications after noncardiothoracic surgery: systematic review for the American College of Physicians. Ann Intern Med. 2006;144:596–608. 16618957 10.7326/0003-4819-144-8-200604180-00011

[39] Orman J, Westerdahl E. Chest physiotherapy with positive expiratory pressure breathing after abdominal and thoracic surgery: a systematic review. Acta Anaesthesiol Scand. 2010;54:261–267. 19878100 10.1111/j.1399-6576.2009.02143.x

[40] Westerdahl E, Lindmark B, Eriksson T, Friberg O, Hedenstierna G, Tenling A. Deep-breathing exercises reduce atelectasis and improve pulmonary function after coronary artery bypass surgery. Chest. 2005;128:3482–3488. 16304303 10.1378/chest.128.5.3482

[41] Chumillas S, Ponce JL, Delgado F, Viciano V, Mateu M. Prevention of postoperative pulmonary complications through respiratory rehabilitation: a controlled clinical study. Arch Phys Med Rehabil. 1998;79:5–9. 9440408 10.1016/s0003-9993(98)90198-8

[42] Canet J, Gallart L, Gomar C, et al. Prediction of postoperative pulmonary complications in a population-based surgical cohort. Anesthesiology. 2010;113:1338–1350. 21045639 10.1097/ALN.0b013e3181fc6e0a

[43] Lindberg P, Gunnarsson L, Tokics L, et al. Atelectasis and lung function in the postoperative period. Acta Anaesthesiologica Scandinavica. 1992;36:546–553. 1514340 10.1111/j.1399-6576.1992.tb03516.x

[44] Svensson-Raskh A, Schandl A, Holdar U, Fagevik Olsén M, Nygren-Bonnier M. I have everything to win and nothing to lose: patient experiences of mobilization out of bed immediately after abdominal surgery. Phys Ther. 2020;100:2079–2089. 32941610 10.1093/ptj/pzaa168 PMC7720638

