# Supplementary material for: Mobilization Started Within 2 Hours After Abdominal Surgery Improves Peripheral and Arterial Oxygenation: A Single-Center Randomized Controlled Trial
Source: Phys Ther. 2021 Mar 20;101(5):pzab094. doi: 10.1093/ptj/pzab094 (PMC8136304; doi:10.1093/ptj/pzab094)
Supplement: SUPPLEMENTARY_1_pzab094 [file supplementary_1_pzab094.docx]

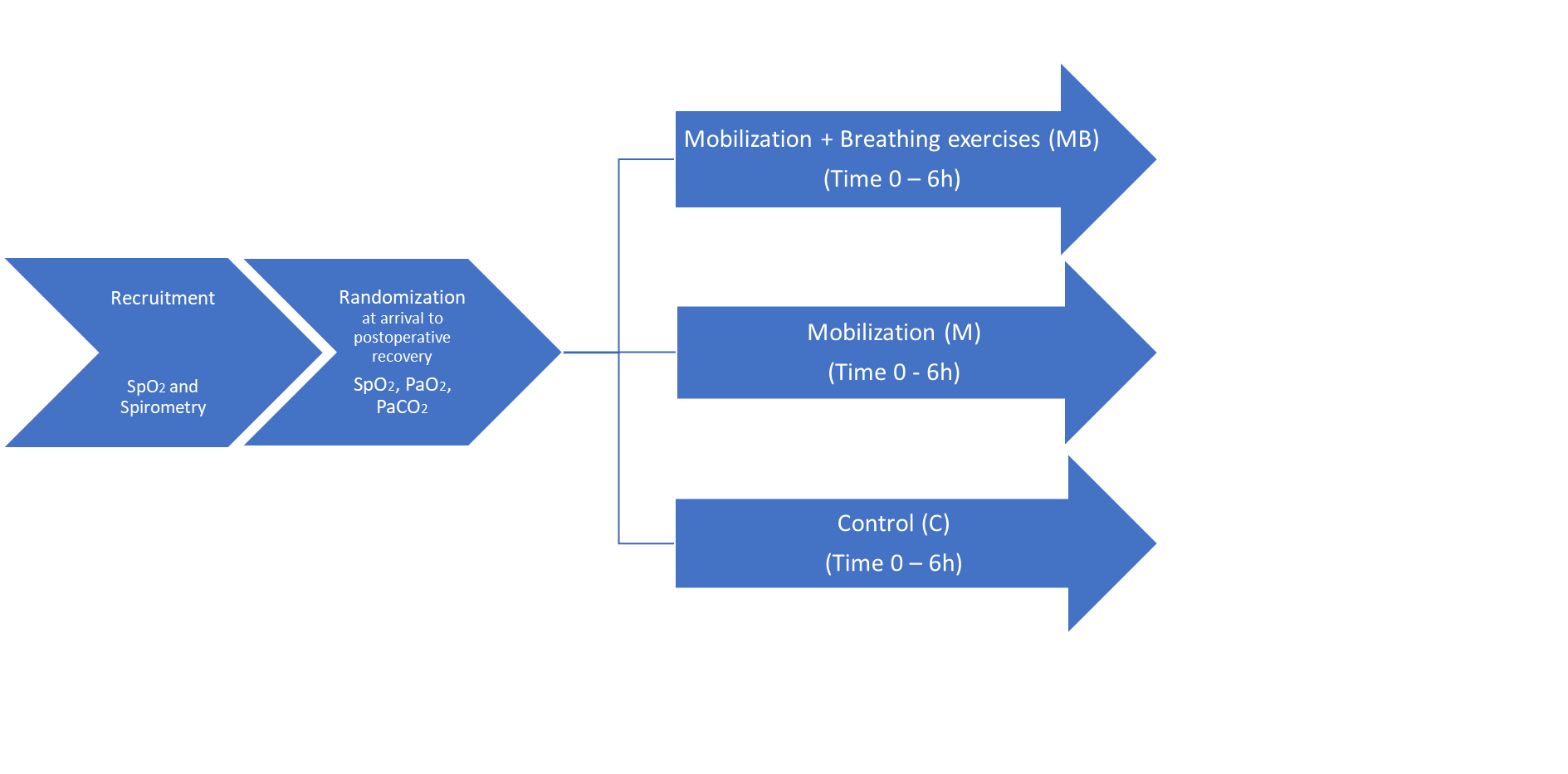


**Supplementary 1. Figure 3.** Study timeline of the procedures during the trial, from recruitment, to interventions to final assessment.

SpO2, PaO2, PaCO2 every hour. From arrival until end of trial when discharge from the recovery unit or for a maximum of 6 hours.

-2 weeks POD0 0h 1h 2h 3h 4h 5h 6h POD1 +2 weeks

2 weeks after surgery

Assessment in Medical records for Pneumonia

POD 1

Spirometry
