# Supplementary material for: Mobilization Started Within 2 Hours After Abdominal Surgery Improves Peripheral and Arterial Oxygenation: A Single-Center Randomized Controlled Trial
Source: Phys Ther. 2021 Mar 20;101(5):pzab094. doi: 10.1093/ptj/pzab094 (PMC8136304; doi:10.1093/ptj/pzab094)
Supplement: SUPPLEMENTARY_2_pzab094 [file supplementary_2_pzab094.docx]

**SUPPLEMENTARY 2a. Primary and secondary outcomes in the intention-to-treat population (n=214), by treatment group.**

| **Variable** | | **Raw mean scores (95% CIs) in the ITT population** | | | | | |
| --- | --- | --- | --- | --- | --- | --- | --- |
|  | Time hours | n=73 | **Mobilization and Breathing exercises** | n=76 | **Mobilization** | n=65 | **Control** |
|  |  |  |  |  |  |  |  |
| **SpO_2_** | Pre-op | n=73 | 97.2 (96.2 to 98.1) | n=76 | 97.2 (96.3 to 98.1) | n=65 | 97.6 (96.6 to 98.6) |
|  | 0 | n=72 | 92.9 (91.9 to 93.8) | n=76 | 92.0 (91.1 to 92.9) | n=64 | 93.0 (92.0 to 94.0) |
|  | 1 | n=64 | 93.6 (92.6 to 94.6) | n=71 | 92.8 (91.9 to 93.7) | n=65 | 93.0 (92.0 to 94.0) |
|  | 2 | n=67 | 95.0 (94.0 to 95.9) | n=69 | 94.3 (93.4 to 95.2) | n=60 | 93.3 (92.3 to 94.3) |
|  | 3 | n=59 | 95.5 (94.5 to 96.5) | n=62 | 95.1 (94.1 to 96.1) | n=48 | 93.3 (92.2 to 94.4) |
|  | 4 | n=45 | 95.8 (94.6 to 96.9) | n=43 | 92.9 (91.7 to 94.0) | n=32 | 93.2 (91.9 to 94.5) |
|  |  |  |  |  |  |  |  |
| **PaO_2_** | 0 | n=64 | 10.04 (9.57 to 10.51) | n=72 | 9.82 (9.42 to 10.21) | n=63 | 10.51 (10.08 to 10.94) |
|  | 1 | n=57 | 10.46 (10.14 to 10.78) | n=66 | 10.27 (9.96 to 10.55) | n=59 | 10.08 (9.77 to 10.38) |
|  | 2 | n=60 | 10.84 (10.48 to 11.20) | n=65 | 10.71 (10.37 to 11.05) | n=54 | 10.18 (9.82 to 10.55) |
|  | 3 | n=54 | 11.22 (10.81 to 11.63) | n=58 | 11.20 (10.81 to 11.58) | n=44 | 10.11 (9.68 to 10.54) |
|  | 4 | n=41 | 11.28 (10.86 to 11.70) | n=37 | 10.84 (10.41 to 11.27) | n=32 | 9.87 (9.41 to 10.33) |
|  |  |  |  |  |  |  |  |
| **PaCO_2_** | 0 | n=64 | 5.58 (5.43 to 5.74) | n=72 | 5.63 (5.48 to 5.77) | n=63 | 5.54 (5.35 to 5.72) |
|  | 1 | n=57 | 5.41 (5.29 to 5.54) | n=66 | 5.51 (5.40 to 5.62) | n=59 | 5.43 (5.30 to 5.55) |
|  | 2 | n=60 | 5.21 (5.09 to 5.33) | n=65 | 5.23 (5.12 to 5.34) | n=54 | 5.43 (5.31 to 5.55) |
|  | 3 | n=54 | 5.12 (5.00 to 5.24) | n=58 | 5.20 (5.08 to 5.31) | n=44 | 5.25 (5.12 to 5.38) |
|  | 4 | n=41 | 5.02 (4.89 to 5.16) | n=37 | 5.22 (5.08 to 5.36) | n=32 | 5.15 (5.00 to 5.29) |

**SUPPLEMENTARY TABLE 2b. Primary and secondary outcomes in the per-protocol population (n=201), by treatment group.**

| **Variable** | | **Raw mean scores (95% CIs) in the PP population** | | | | | |
| --- | --- | --- | --- | --- | --- | --- | --- |
|  | Time hours | n=68 | **Mobilization and Breathing exercises** | n=69 | **Mobilization** | n=64 | **Control** |
|  |  |  |  |  |  |  |  |
| **SpO_2_** | Pre-op | n=68 | 97.2 (96.6 to 97.8) | n=69 | 97.3 (96.7 to 97.9) | n=64 | 97.5 (96.9 to 98.1) |
|  | 0 | n=67 | 92.9 (92.3 to 93.5) | n=69 | 92.1 (91.5 to 92.7) | n=63 | 93.0 (92.4 to 93.6) |
|  | 1 | n=60 | 93.6 (92.9 to 94.2) | n=66 | 93.1 (92.5 to 93.7) | n=64 | 93.0 (92.4 to 93.6) |
|  | 2 | n=65 | 95.1 (94.5 to 95.7) | n=67 | 94.6 (94.0 to 95.2) | n=60 | 93.2 (92.6 to 93.9) |
|  | 3 | n=57 | 95.5 (94.9 to 96.2) | n=60 | 95.3 (94.7 to 96.0) | n=48 | 93.3 (92.6 to 93.9) |
|  | 4 | n=43 | 95.9 (95.2 to 96.7) | n=42 | 95.6 (94.9 to 96.3) | n=32 | 93.2 (92.4 to 94.0) |
|  |  |  |  |  |  |  |  |
| **PaO_2_** | 0 | n=64 | 10.04 (9.57 to 10.51) | n=66 | 9.82 (9.42 to 10.21) | n=63 | 10.51 (10.08 to 10.94) |
|  | 1 | n=57 | 10.38 (10.06 to 10.70) | n=66 | 10.34 (10.05 to 10.63) | n=59 | 10.05 (9.75 to 10.35) |
|  | 2 | n=59 | 10.86 (10.49 to 11.22) | n=63 | 10.81 (10.47 to 11.16) | n=54 | 10.16 (9.80 to 10.53) |
|  | 3 | n=53 | 11.23 (10.81 to 11.65) | n=56 | 11.31 (10.91 to 11.70) | n=44 | 10.09 (9.66 to 10.52) |
|  | 4 | n=40 | 11.42 (10.96 to 11.89) | n=36 | 11.10 (10.62 to 11.58) | n=32 | 9.87 (9.36 to 10.37) |
|  |  |  |  |  |  |  |  |
| **PaCO_2_** | 0 | n=64 | 5.61 (5.47 to 5.78) | n=66 | 5.63 (5.35 to 5.72) | n=63 | 5.54 (5.45 to 5.77) |
|  | 1 | n=57 | 5.41 (5.28 to 5.54) | n=66 | 5.52 (5.40 to 5.64) | n=59 | 5.54 (5.42 to 5.66) |
|  | 2 | n=59 | 5.20 (5.08 to 5.32) | n=63 | 5.21 (5.10 to 5.33) | n=54 | 5.43 (5.31 to 5.55) |
|  | 3 | n=53 | 5.11 (4.98 to 5.23) | n=56 | 5.18 (5.06 to 5.30) | n=44 | 5.26 (5.13 to 5.39) |
|  | 4 | n=40 | 5.01 (4.87 to 5.15) | n=36 | 5.21 (5.07 to 5.35) | n=32 | 5.15 (5.01 to 5.30) |

Adjustment for multiple comparisons: Bonferroni

Covariate at SpO_2_: Age. Covariate at PaO_2_: Age and PaO_2_ at time 0. Covariate at PaCO_2_: Age and PaCO_2_ at time 0.
