# Supplementary material for: Mobilization Started Within 2 Hours After Abdominal Surgery Improves Peripheral and Arterial Oxygenation: A Single-Center Randomized Controlled Trial
Source: Phys Ther. 2021 Mar 20;101(5):pzab094. doi: 10.1093/ptj/pzab094 (PMC8136304; doi:10.1093/ptj/pzab094)
Supplement: SUPPLEMENTARY_3_pzab094 [file supplementary_3_pzab094.docx]

**Supplementary 3.** Secondary outcomes in the per protocol population (n=201), by treatment group.

| Mean scores (95% CIs) and mean score differences MD (95% CIs) | | | | | |
| --- | --- | --- | --- | --- | --- |
| Secondary outcomes |  | **Mobilization and Breathing exercises**  **(n=68)** | **Mobilization**  **(n=69)** | **Control**  **(n=64)** | **P value** |
| FVC | Preoperatively | 3.37 (3.15-3.58) | 3.28 (3.01-3.48) | 3.07 (2.85-3.29) |  |
|  | POD 1 | 2.84 (2.61-3.07) | 2.72 (2.50-2.95) | 2.48 (2.24-2.72) | .88 |
|  | MD (95% CI) | -0.53* (-0.69 to -0.37) | -0.55* (-0.71 to -0.39) | -0.59* (-0.76 to -0.42) |  |
|  |  |  |  |  |  |
| FEV_1_ | Preoperatively | 2.53 (2.36-2.70) | 2.46 (2.30-2.63) | 2.38 (2.21-2.55) |  |
|  | POD 1 | 2.17 (1.98-2.35) | 2.10 (1.92-2.28) | 1.94 (1.76-2.13) | .72 |
|  | MD (95% CI) | -0.36* (-0.49 to -0.23) | -0.36* (-0.49 to -0.23) | -0.43* (-0.57 to -0.29) |  |
|  |  |  |  |  |  |
| PEF | Preoperatively | 378.77 (347.82-409.73) | 381.70 (351.68-411.72) | 363.44 (332.33-394.55) |  |
|  | POD 1 | 313.87 (279.99-347.75) | 313.04 (279.75-346.33) | 300.17 (265.18-335.16) | .96 |
|  | MD (95% CI) | -64.9* (-91.38 to -38.43) | -68.66* (-94.93 to -42.40) | -63.27* (-91.29 to -35.25) |  |
|  |  |  |  |  |  |
| FEV_1_/FVC | Preoperatively | 77.18 (75.11-79.24) | 77.56 (75.56-79.56) | 78.40 (76.33-80.48) |  |
|  | POD 1 | 78.58 (76.27-80.89) | 77.23 (74.96-79.51) | 79.74 (77.35-82.14) | .4 |
|  | MD (95% CI) | 1.4 (-0.62 to 3.43) | -0.33 (-2.33 to 1.68) | 1.34 (-0.79 to 3.48) |  |
|  | | | | |  |
| Respiratory insufficiency | | 13 (19%) | 19 (27%) | 16 (25%) | .5 |
| Pneumonia | | 0 (0%) | 2 (3%) | 5 (8%) | .03 |
| Length of stay at  postoperative recovery, hours | | 12 (10) | 9 (7) | 9 (7) | .63 |
| Length of stay at hospital, days | | 5 (3) | 4 (3) | 4 (3) | .42 |

Data are n (%) or mean (SD) unless otherwise indicated. POD1=postoperative day one after surgery; FVC (Forced Vital Capacity L); FEV_1_ (Forced Expiratory Volume in one second); PEF (Peak Expiratory Flow L/min); FEV_1_/FVC (%) * represents statistically significant difference (P< .05) within the group between preoperatively assessment and POD1. Respiratory insufficiency (SpO_2_<90% and/or PaO_2_<8kPa and/or PaCO_2_≥6.5kPa (at any point) from 1 hour after arrival at postoperative recovery unit until discharge. Pneumonia means by a physician registered in the medical record and within 2 weeks of surgery. P value represents the difference between the groups P< .05
